# Intrinsic and Synaptic Properties Shaping Diverse Behaviors of Neural Dynamics

**DOI:** 10.3389/fncom.2020.00026

**Published:** 2020-04-21

**Authors:** Lingling An, Yuanhong Tang, Doudou Wang, Shanshan Jia, Qingqi Pei, Quan Wang, Zhaofei Yu, Jian K. Liu

**Affiliations:** ^1^School of Computer Science and Technology, Xidian University, Xi'an, China; ^2^National Engineering Laboratory for Video Technology, Department of Computer Science and Technology, Peking University, Beijing, China; ^3^Centre for Systems Neuroscience, Department of Neuroscience, Psychology and Behaviour, University of Leicester, Leicester, United Kingdom

**Keywords:** neural dynamic modeling, stability analysis, rebound firing, synaptic dynamics, dynamical system

## Abstract

The majority of neurons in the neuronal system of the brain have a complex morphological structure, which diversifies the dynamics of neurons. In the granular layer of the cerebellum, there exists a unique cell type, the unipolar brush cell (UBC), that serves as an important relay cell for transferring information from outside mossy fibers to downstream granule cells. The distinguishing feature of the UBC is that it has a simple morphology, with only one short dendritic brush connected to its soma. Based on experimental evidence showing that UBCs exhibit a variety of dynamic behaviors, here we develop two simple models, one with a few detailed ion channels for simulation and the other one as a two-variable dynamical system for theoretical analysis, to characterize the intrinsic dynamics of UBCs. The reasonable values of the key channel parameters of the models can be determined by analysis of the stability of the resting membrane potential and the rebound firing properties of UBCs. Considered together with a large variety of synaptic dynamics installed on UBCs, we show that the simple-structured UBCs, as relay cells, can extend the range of dynamics and information from input mossy fibers to granule cells with low-frequency resonance and transfer stereotyped inputs to diverse amplitudes and phases of the output for downstream granule cells. These results suggest that neuronal computation, embedded within intrinsic ion channels and the diverse synaptic properties of single neurons without sophisticated morphology, can shape a large variety of dynamic behaviors to enhance the computational ability of local neuronal circuits.

## 1. Introduction

The vast majority of neurons in the neuronal systems of the brain have a complex morphology with a tree-like structure of the dendritic field. Each spine on the dendrite represents a synaptic contact that receives its input from a presynaptic neuron. The integration of these tremendous synapses is computationally complicated with dendritic non-linearity (Poirazi et al., 2003). However, there are other neurons with a relatively simple dendritic structure, but still conducting complex computation, as the important factors for single neuronal dynamics consist of a series of interactions between intrinsic and synaptic dynamics (Abbott and Regehr, 2004; Harvey et al., 2009; Torres et al., 2019).

The cerebellum, as one of the most stereotyped brain areas, has been traditionally suggested to play an essential role in motor control (Zeeuw et al., 1997). In recent years, a large body of studies has shown that the cerebellum is also involved in the processing of higher cognition (Ito, 2008; Strick et al., 2009; Tsai et al., 2012; Wagner et al., 2017; Bostan and Strick, 2018; Raymond and Medina, 2018). From a classical viewpoint, the cerebellum combines all sensory information about the body and outside environment to compute a series of motor sequences for controlling body movements and correcting the body position in space. For the granular layer of the cerebellum, all of these computations are started from mossy fibers (MFs) as outside inputs and ended with granule cells (GCs) as outputs.

**Figure 1:**
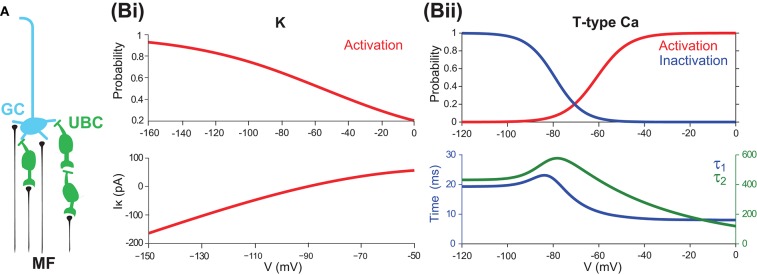
UBCs as relay cells between MFs and GCs **(A)** A typical GC receives four presynaptic inputs from MFs and/or UBCs. **(B)** Channel properties of mGluR2-dependent rectifier K and T-type Ca currents. **(Bi)** mGluR2-dependent rectifier K (*I*_*K*_) steady-state activation curve (top) and its current as a function of membrane potential (bottom). **(Bii)** T-type Ca steady-state activation and inactivation curves (top) and voltage sensitive time constants τ_1_ and τ_2_. Note that τ_2_ is much larger than τ_1_.

In the granular layer, there is a high density of compact GCs. On average, each GC only receives four presynaptic inputs. However, one of the unique features of the connection between MF inputs and well-studied GCs is the presence of an additional type of neuron, termed a unipolar brush cell (UBC) (Mugnaini et al., 1997), that constitutes an independent relay line for external MF signals. Therefore, UBCs have been suggested as internal MFs playing the important role of determining how the information from external MFs is transferred and encoded by the cerebellar cortex (Mugnaini et al., 1994, 2011; Jaarsma et al., 1995; Kinney et al., 1997; Diño et al., 1999; Nunzi et al., 2002; Ann et al., 2014; Stijn and Zeeuw, 2014; Carolina and Trussell, 2015; Zampini et al., 2016). Information flows into the granular layer through external MFs and is then relayed by UBCs as a version of internal MFs, such that GCs receive external MFs and transcoded UBCs. Besides, there are cascaded connections between UBCs formed as internal transcode lines from external MFs to GCs, as illustrated in Figure 1A, where the evidence from experimental and computational studies shows that UBCs make a significant contribution to transfer and relay of external MF activity for downstream GCs and Purkinje cells (Ann et al., 2014; Carolina and Trussell, 2015; Zampini et al., 2016).

The unique feature of UBCs is that they are glutamatergic interneurons receiving a single excitatory synapse from MF and several inhibitory buttons from Golgi cells on their short dendritic brush (Mugnaini et al., 2011). Experimental studies have demonstrated that the unusually large MF-UBC synapse may entrap glutamate in the synaptic cleft that can prolong the excitatory postsynaptic currents mediated by various receptors in UBCs following MF stimulation (Rossi et al., 1995; Kinney et al., 1997; Morin et al., 2001). Together with inhibition from Golgi cells, there is a large variety of dynamic properties of the UBCs from pure excitation to pure inhibition, and a mix of both (Carolina and Trussell, 2015; Zampini et al., 2016).

The variation of biophysical components in UBCs enables them to exhibit a rich range of dynamics. On the one hand, the strong feedforward excitation encoded by UBCs can amplify the external MF signal as an ON signal for downstream GCs. On the other hand, the inhibition mediated by UBCs plays the role of an OFF signal to rectify the inputs (Carolina and Trussell, 2015; Zampini et al., 2016). Together with other mixed excitation and inhibition from UBCs, the input-output relationship of the granular layer can be modulated in a highly dynamical way (Ann et al., 2014). Various synaptic properties of UBCs make them send fast and slow types of excitation and inhibition down to the GCs (Stijn and Zeeuw, 2014; Zampini et al., 2016). At the same time, the intrinsic properties of UBCs are also determinant factors for their computational properties and shape their synaptic responses and firing patterns (Subramaniyam et al., 2014).

Despite the granular layer being relatively well-understood, the dynamics and functional properties of UBCs remain unclear due to the complex combinations of ion channels and synaptic receptors. In this study, we investigate the relationship between the activity displayed by UBCs and underlying voltage-dependent ion channels and various types of synaptic receptors with a modeling approach. We use a full model of a UBC with a number of ion channels and a reduced minimal model with essential T-type calcium and rectifier potassium channels to study the intrinsic properties of UBC and clarify the role of these two channels in shaping the dynamics of UBC response while maintaining the stability of the UBC resting membrane potential. Rebound firing after inhibition offset and low-frequency resonance are also exhibited by both versions of the model. Finally, the UBC firing patterns determined by intrinsic properties are also regulated by different types of synaptic receptors, such that UBCs can show a large variation of temporal dynamics, as observed in experiments.

## 2. Methods

### 2.1. Full UBC Model

Here, the full UBC model is a model with all essential ion channels to generate experimental observations, although a more detailed model was derived previously (Subramaniyam et al., 2014). The UBC was modeled as an integration-and-fire neuron but with additional T-type calcium (Ca) (*I*_*T*_), L-type Ca (*I*_*L*_), and mGluR2-dependent rectifier potassium K (*I*_*K*_) current, as well as h-type (*I*_*h*_) current. The membrane potential *V* is changed with time according to the following equation when *V* < *V*_*thr*_,

(1) $$\begin{array}{l} C\left(dV/dt\right)=-g_{l}\left(V-E_{l}\right)-g_{AHP}\left(t\right)\left(V-E_{K}\right)-I_{T}-I_{K}-I_{h}\\ \;-\;I_{L}-I_{input}, \end{array}$$

where *C* = 20 pF is the cell capacitance, *g*_*l*_ = 1 nS and *E*_*l*_ = −67 mV represent the conductance and resting potential of the leak current, and *I*_*input*_ is the input current. When the threshold *V*_*thr*_ = −50 mV is reached at the spiking time *t* = *t*_*spk*_, *V* is set to *V*_*peak*_ = 40 mV for a duration of the spike of τ_*dur*_ = 1 ms. Then, after the spike at *t* = *t*_*spk*_ + τ_*dur*_, the repolarizing potential is set to *V*_*reset*_ = −67 mV, and the after hyperpolarization conductance (*g*_*AHP*_) is activated with *E*_*K*_ = −90 mV. *g*_*AHP*_ is a dynamical conductance changed as follows:

(2) $$\begin{array}{l} dg_{AHP}/dt=-g_{AHP}/\tau_{AHP}+{ḡ}_{AHP}\delta \left(t-t_{spk}-\tau_{dur}\right), \end{array}$$

where τ_*AHP*_ = 2 ms, and the δ function is used to add an increment ḡ_*AHP*_ = 1 nS where a spike occurs. The refractory period is set as τ_*ref*_ = 2 ms.

Looking more closely at these channels, there are two channels that are particularly interesting. The first is the rectifier K channel, which plays an important role in maintaining the stability of the resting membrane potential, controlling excitability, and shaping the initial depolarization (Chung et al., 2010). For UBCs, there is a specific type of K channel, the mGluR2-dependent rectifier type, which can be modeled based on experimental data (Zampini et al., 2016) as

(3) $$\begin{array}{l} \begin{array}{c} \;I_{K}=g_{K}m_{\infty ,K}\left(V-E_{K}\right)\\ m_{\infty ,K}=1/\left(1+\text{exp}\left(\left(V+55\right)/40.5\right)\right) \end{array} \end{array}$$

where *g*_*K*_ is the conductance, which is a free parameter, and *E*_*K*_ = −90 mV is the reversal potential. *m*_∞, *K*_ represents the activation state. Figure 1Bi shows the typical behavior, activation curve, and voltage-current curve of this channel followed by the model. Half-point activation is reached at -55 mV for the rectifier K channel. Note that this current has a fast and instantaneous activation time constant, such that the output current at the given membrane potential can be computed directly by this steady state, as an input-output, or voltage-current, mapping as in Figure 1Bi (bottom).

The other channel of interest is the fast-inactivating T-type Ca current, which produces low-threshold spikes, triggering the high-frequency bursts, and generating powerful Ca transients in the brush and soma (Diana et al., 2007; Birnstiel et al., 2009). The T-type Ca current can be modeled as

(4) $$\begin{array}{l} \begin{array}{c} \;I_{T}=g_{T}m_{\infty ,T}h\left(V-E_{ca}\right)\\ m_{\infty ,T}=1/\left(1+exp\left(-\left(V+61\right)/7\right)\right) \end{array} \end{array}$$

where *g*_*T*_ is the conductance, which is a free parameter, *E*_*Ca*_ = 120 mV is the reversal potential, *m*_∞, *T*_ represents the activation state, and *h* is the inactivation described below.

The T-type Ca current inactivates rapidly during maintained depolarization yet recovers slowly from inactivation. To reconcile these observations, a two-step kinetic scheme is used for the inactivation gate. All the time constants in this scheme are voltage-dependent. To account for these effects, we consider a model that has two closed states (*C*_1_ and *C*_2_) and one open (*O*) state (Wang et al., 1991),

(5) $$\begin{array}{l} C_{2}\overset{\alpha_{2}}{\underset{\beta_{2}}{⇌}}C_{1}\overset{\alpha_{1}}{\underset{\beta_{1}}{⇌}}O. \end{array}$$

Considerable low transition rates into and out of the deep closed state *C*_2_ provide the mechanism for the slower state of inactivation. *h*, *d*, and *s* are defined as the fractions of inactivation gates in the states *O*, *C*_1_, and *C*_2_, respectively. Since *h*+*d*+*s* = 1, the equations for *h* and *s* are

(6) $$\begin{array}{l} \begin{array}{c} dh/dt=\alpha_{1}\left(1-h-s\right)-\beta_{1}h\\ ds/dt=\beta_{2}\left(1-h-s\right)-\alpha_{2}s \end{array} \end{array}$$

In this model, there are four rate coefficient kinetics: *K*_1_, *K*_2_, τ_1_, and τ_2_, or equivalently,

(7) $$\begin{array}{l} \begin{array}{c} K_{1}=\beta_{1}/\alpha_{1},K_{2}=\beta_{1}/\alpha_{2}\\ \;\tau_{1}=1/\left(\alpha_{1}+\beta_{1}\right),\tau_{2}=1/\left(\alpha_{2}+\beta_{2}\right) \end{array} \end{array}$$

Assuming *K*_1_ = *K*_2_, *h*_∞_ = 1/(1 + *K* + *K*^2^). One can solve three rate coefficients by using the following experimental estimation (Diana et al., 2007),

(8) $$\begin{array}{l} h_{\infty }=1/\left(1+\text{exp}\left(\left(V+79.2\right)/6.2\right)\right)\\ \;\tau_{1}=8+\left(211.4+\text{exp}\left(\left(V+111.2\right)/5\right)\right)\\ \;/\;\left(18.7\left(1+\text{exp}\left(\left(V+82\right)/3.5\right)\right)\right)\\ \;\tau_{2}=8+\left(211.4+\text{exp}\left(\left(V+111.2\right)/5\right)\right)\\ \;/\;\left(0.5\left(1+\text{exp}\left(\left(V+82\right)/4.5\right)\right)\right) \end{array}$$

With this model, one can analyze the steady state of membrane potential for the T-type Ca as shown in Figure 1Bii with steady-state activation and inactivation curves fitted with a Boltzmann function. The threshold for T-type Ca (measured as the point of 10% activation) is −76 mV, and half-activation is reached at −61 mV. Half-point inactivation is reached at −79 mV. These values are consistent with those experimental values (Edward, 2003; Diana et al., 2007). Furthermore, the overlap of activation and inactivation curves suggests that the full steady-state inactivation is not achieved between −71 and −50 mV, thus giving the UBC a tonic inward current and Ca influx at subthreshold potentials, similar to what has been reported in thalamocortical relay cells (Vincenzo et al., 2005).

In addition, we also consider two other typical ion channels. One is the L-type current *I*_*L*_ modeled according to experimental data as (Diana et al., 2007)

(9) $$\begin{array}{l} \begin{array}{c} \;I_{L}=g_{L}m_{\infty ,L}\left(V-E_{Ca}\right)\\ m_{\infty ,L}=1/(\left(1+\text{exp}\left(-\left(V+28.6\right)/8.4\right)\right) \end{array} \end{array}$$

with *g*_*L*_ = 0.5 nS and *E*_*Ca*_ = 120 mV. The other is the h-type current *I*_*h*_ modeled according to experimental data as (Diana et al., 2007)

(10) $$\begin{array}{l} \;I_{h}=g_{h}m\left(V-E_{h}\right)\\ dm/dt=\left(m_{\infty ,h}-m\right)/\tau_{m}\\ \;m_{\infty ,h}=\frac{1}{\left(1+\text{exp}\left(\left(V+105\right)/5.5\right)\right)}\\ \;\tau_{m}=100+90\\ \;/\left(\text{exp}\left(\left(V+46.4\right)/109.3\right)+\text{exp}\left(-\left(V+71.6\right)/13\right)\right), \end{array}$$

where *g*_*h*_ = 1.5 nS and *E*_*h*_ = −40 mV.

### 2.2. Minimal UBC Model for Stability Analysis

Given the full model of UBC as above, note that the intrinsic currents *I*_*T*_ and *I*_*K*_ play a dominant role in maintaining the resting potential *V*_*rest*_ and generating responses for subthreshold input. To study the functional role of the T-type Ca channel and rectifier K channel in shaping the intrinsic dynamics of UBC membrane potential, we can simplify the full model as follows. First, one can ignore all other intrinsic currents except *I*_*l*_, *I*_*T*_, and *I*_*K*_. Then, for the *I*_*T*_ current, considering the relationship between the two time constants such that τ_2_ ≫ τ_1_, as shown in Figure 1Bii (bottom), we can further simplify the *I*_*T*_ component to ignore the slow time constant such that $C_{1}\overset{\alpha_{1}}{\underset{\beta_{1}}{⇌}}O.$ In this way, one can obtain a reduced minimal UBC model as a two-variable dynamical system,

(11) $$\begin{array}{l} C\left(dV/dt\right)=-g_{l}\left(V-E_{l}\right)-g_{T}m_{\infty ,T}h\left(V-E_{Ca}\right)\\ \;-\;g_{K}m_{\infty ,K}\left(V-E_{K}\right)\\ \;dh/dt=\left(h_{\infty }-h\right)/\tau_{1}. \end{array}$$

To consider the stability of the steady state of membrane potential at *V*_*rest*_, we explored a wide range of two parameters, *g*_*T*_ and *g*_*K*_, while keeping the other parameters fixed. For this two-variable dynamical system, denote

(12) $$\begin{array}{l} F\left(V,h\right)=-g_{l}\left(V-E_{l}\right)-g_{T}m_{\infty ,T}h\left(V-E_{Ca}\right)\\ \;-\;g_{K}m_{\infty ,K}\left(V-E_{K}\right)\\ G\left(V,h\right)=\left(h_{\infty }-h\right)/\tau_{1} \end{array}$$

The stability of *V*_*rest*_ is then obtained by calculating eigenvalues of the Jacobian matrix evaluated at *V* = *V*_*rest*_:

(13) $$\begin{array}{l} {\left(\begin{array}{cc} F_{V} & F_{h}\\ G_{V} & G_{h} \end{array}\right)}_{V=V_{rest}} \end{array}$$

The signs of the real part of eigenvalues determine the stability: a positive (negative) sign means *V* = *V*_*rest*_ is unstable (stable). The bifurcation points are where the sign changes.

### 2.3. UBC Synaptic Dynamics

Experimental studies have found that synaptic receptors expressed in UBCs are heterogeneous, with significant variation (Stijn and Zeeuw, 2014; Carolina and Trussell, 2015; Zampini et al., 2016), so that UBCs can express excitatory AMPA (α-amino-3-hydroxy-5-methyl-4-isoxazolepropionic acid), NMDA (N-methyl-D-aspartate), and mGluR1 (metabotropic glutamate receptor type 1) and inhibitory mGluR2 (metabotropic glutamate receptor type 2) receptors. There is also a slow spillover excitatory AMPA current (Zampini et al., 2016). Therefore, we modeled postsynaptic currents in a UBC with fast AMPA (AMPAf), slow spillover AMPA (AMPAsl), NMDA, and mGluR1, as well as mGluR2 dynamics:

(14) $$\begin{array}{l} \begin{array}{c} I_{MF-UBC}=g_{AMPAf}r\left(t\right)V+g_{AMPAsl}r\left(t\right)V\\ \;+g_{NMDA}{\left(1-\left(V+25\right)/12.5\right)}^{-1}r\left(t\right)V\\ \;+g_{mGluR1}r\left(t\right)V+g_{mGluR2}r\left(t\right)\left(V+E_{K}\right) \end{array} \end{array}$$

The gating variable *r*(*t*) for all types of synapses was modeled as:

(15) $$\begin{array}{l} \begin{array}{c} dr/dt=-r/\tau_{decay}+\alpha s\left(1-r\right)\\ ds/dt=-s\tau_{rise}+Ru\underset{k}{\sum }\delta \left(t-t_{spk}\right), \end{array} \end{array}$$

where the value of α was chosen so as to make the peak value of *r* as *U* induced by the first spike. In all synapses except mGluR1 and mGluR2, *Ru* was induced for short-term plasticity, which was modeled as previously (Markram et al., 1998; Izhikevich, 2003):

(16) $$\begin{array}{l} \begin{array}{c} dR/dt=\left(1-R\right)/\tau_{rec}-uR\delta \left(t-t_{n}\right)\\ du/dt=\left(U-u\right)/\tau_{fac}+U\left(1-u\right)\delta \left(t-t_{n}\right), \end{array} \end{array}$$

where *R*(*u*) is the short-term depression (facilitation) variable with the time constant τ_*rec*_ (τ_*fac*_) and subjected to the pulsed decrease *uR* [increase *U*(1−*u*)] due to the *n*-th spike. The cumulative synaptic efficacy at any time is the product *Ru* that is incorporated into single synaptic dynamics. In summary, all synaptic parameters are listed in Table 1. All the simulations were done in MATLAB.

**Table 1:** Parameters of all synaptic receptors together with short-term plasticity for UBC models.

	**Strength**	**Synaptic dynamics**			**Short-term plasticity**		
**Receptor**	***g*_*peak*_ (nS)**	**α (1/ms)**	**τ_*rise*_ (ms)**	**τ_*decay*_ (ms)**	**U**	**τ_*rec*_ (ms)**	**τ_*fac*_ (ms)**
AMPAf	2.3	3	0.4	2	0.5	100–1,000	12
AMPAsl	0.35	0.015	163	504	0.5	400–4,000	12
NMDA	1.15	0.35	5	100	0.5	100–1,000	12
mGluR1	1	0.01	292	2,580	0.03	-	-
mGluR2	2	0.01	150	500	0.1	-	-

## 3. Results

### 3.1. Stability of the Resting Membrane Potential in UBCs

UBCs are relay cells between external MF inputs and GCs (Ann et al., 2014; Zampini et al., 2016), as illustrated in Figure 1A. Besides, the axons of UBCs themselves form a version of internal MFs to connect with other UBCs and GCs and establish an intrinsic relay network that can synchronize, amplify, and relay MFs inputs to downstream GCs (Birnstiel et al., 2009; Zampini et al., 2016). Moreover, the intrinsic properties of UBCs are important determinant factors for the computational performance of this relay network (Diana et al., 2007). To study these properties, we set up a full UBC model with all essential ion channels to generate experimental observations. The model is based on an integration-and-fire neuron but with additional T-type Ca (*I*_*T*_), L-type Ca (*I*_*L*_), mGluR2-dependent rectifier K (*I*_*K*_), and h-type (*I*_*h*_) ion channel currents (see section Methods). In contrast to a detailed UBC model with all known ion channels and morphology (Subramaniyam et al., 2014), it should be noted that the full model here is simply to include the necessary, but a subset of all, component channels to explain UBC dynamics.

Within the full model above, the intrinsic properties of *I*_*T*_ and *I*_*K*_ play a dominant role in maintaining the resting membrane potential, modulating subthreshold responses as well as spiking activity. To study the functional role of those two channels, we simplified the full model to a reduced minimal UBC model that becomes a simple two-variable dynamical system with a Jacobian matrix to evaluate eigenvalues for stability analysis (see section Methods).

**Figure 2:**
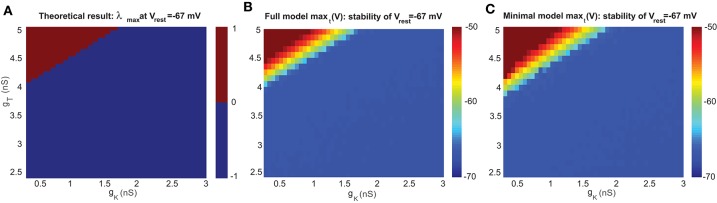
Stability analysis of the resting membrane potential of a UBC. **(A)** Bifurcation diagram based on theoretical analysis of eigenvalue λ at *V*_*rest*_=-67 mV. The upper part of the bifurcation diagram (red) indicates that the UBC is unstable with positive eigenvalue, and the lower part (blue) is stable with a negative one. **(B)** Bifurcation diagram simulated by the full model at *V*_*rest*_=-67 mV. **(C)** Similar to **(B)** but for the minimal model.

The stability of the resting membrane potential is used to study the steady state of the resting membrane potential *V*_*rest*_. As we consider those the two most important channels, we computed Jacobian matrices at a wide range of key parameters; in this case, the conductances of *g*_*T*_ and *g*_*K*_. The sign of the real part of eigenvalues determines the stability: a positive (negative) sign means *V* = *V*_*rest*_ is unstable (stable). The bifurcation points are where the sign changes. Theoretical results with eigenvalues λ are shown in Figure 2 in the parameter space of *g*_*T*_ and *g*_*K*_.

For the steady state of membrane potential, it is the leakage potential *E*_*l*_ in the model that is equivalent to the resting potential *V*_*rest*_ measured in experiments. Experimental observation found that there is no spontaneous firing when the single UBC is recorded by blocking all receptors, which that means *V*_*rest*_ is stable. Figure 2A shows the theoretical bifurcation diagram of the Jacobian matrix evaluated at *V*_*rest*_ = −67 mV, the value measured by experiments (Diana et al., 2007).

To analyze the stability property in detail, we compared the Jacobian matrix of the theoretical analysis with the simulated membrane potentials in the full and minimal models at *E*_*l*_ = −67 mV. With the same set of values for parameters *g*_*T*_ and *g*_*K*_, both versions of the UBC model were simulated for a period of *t*=1 s, and then the maximum value of stimulated membrane potential max_*t*_ (*V*) over this period *t* was obtained for each pair of *g*_*T*_ and *g*_*K*_. The results of max_*t*_ (*V*) are presented as the corresponding bifurcation diagrams in Figure 2B for the full model and Figure 2C for the minimal model. max(*V*) = −50 mV is the threshold for firing a spike in the model, so the membrane potential below this value is in the stable state. The comparison shows that the results of the two models are consistent with the theoretical result in the sense that the values of *g*_*T*_ and *g*_*K*_ are within a similar range for the stable resting potential, while the minimal model shifts the boundary line of stability by a small amount due to ignoring several ion channels in the model. These stability diagrams can give an estimate of the possible parameter values of *g*_*T*_ and *g*_*K*_ for future simulations below.

### 3.2. Rebound Firing in a UBC

Experimental observations show that some types of UBCs have a particular feature of rebound firing after the offset of inhibitory stimulus, which plays a functional role in information timing and encoding (Zampini et al., 2016). Similar to other types of neurons showing rebound firing in the cortex, the low threshold voltage-gated T-type Ca channel is thought to contribute to rebound firing because it can activate and inactivate with relatively little depolarization as shown in Figure 1Bii. It has been suggested that several mechanisms could produce hyperpolarization rebound, which could signal specific patterns of animal behavior (Diana et al., 2007).

**Figure 3:**
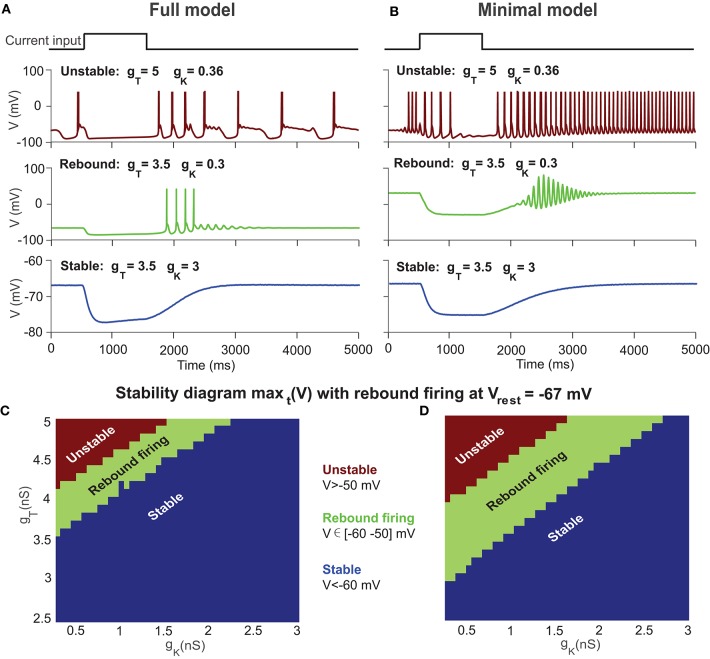
Stability diagram of a UBC with rebound firing at *V*_*rest*_ = −67 mV. **(A)** Examples of UBC response in the full model with different combinations of *g*_*T*_ and *g*_*K*_ for unstable state (top), rebound firing (middle), and stable state (bottom) after the offset of a constant inhibitory current. **(B)** Similar to **(A)** but with the minimal model. **(C)** Stability diagram of the UBC in the full model at *V*_*rest*_ =-67 mV showing three regions of unstable state (max_*t*_ (*V*) >-50 mV), rebound firing (max_*t*_ (*V*) ∈ [-60−50] mV), and stable state (max_*t*_ (*V*) <-60 mV) in the parameter space of *g*_*T*_ and *g*_*K*_. **(D)** Similar to **(C)** but with the minimal model.

A typical property of the T-type current in neurons is to generate rebound firing when an inhibitory current is injected. Figures 3A,B shows a few typical examples of UBC voltage traces simulated with both models at different settings of *g*_*T*_ and *g*_*K*_. Similar to the stability diagram, the firing patterns of the UBC are shaped by ion channels. Higher *g*_*T*_ values set the UBC to an unstable state, and lower values are for the stable state. However, for those values close to the bifurcation boundary, there is a regime of rebound firing after the offset of inhibitory current stimulation.

We measured the maximal value of membrane potentials after stimulation offset and obtained the rebound firing diagram simulated with UBC models by varying *g*_*T*_ and *g*_*K*_, as shown in Figures 3C,D. The theoretical results suggest that the values of *g*_*T*_ and *g*_*K*_ in a UBC should be close to the bifurcation boundary in the stability diagram to generate rebound firing, where *V* ∈ [−60 −50] mV is marked as the rebound firing regime. Within the stable regime of Figure 2, the UBC presents rebound firing activity, as shown in experiments. A typical membrane voltage trace of the full model for rebound firing is shown in Figure 3A (middle) by using the parameter values measured in experiments for the conductance *g*_*T*_ = 3.5 nS and *g*_*K*_ = 0.3 nS. With these values, the UBC does not present spontaneous firing but shows rebound firing with an inhibitory current. We then fix the two parameters at these values for the rest of the paper. These results suggest that the intrinsic T-type Ca channel can profoundly shape the response to MF input and mediate rebound firing in a UBC.

### 3.3. UBC Subthreshold Resonance

Generation of action potentials in neurons depends on how relatively weaker inputs influence the dynamics of subthreshold voltage without firing activity. One typical behavior for subthreshold neuronal dynamics is showing membrane potential resonance, i.e., there there is a maximal subthreshold response at a non-zero frequency (Ostojic et al., 2015). To investigate the subthreshold resonance of a UBC, we injected a sinusoidal zap current with increasing frequency. If there were a significant resonance response at a certain frequency, the membrane potential would be amplified. To obtain the resonance frequency, Fast Fourier Transform (FFT) was used for the voltage response of the UBC to get a spectrum of response amplitude. The frequency at which the maximal amplitude is observed is called the resonance frequency, which is set by a combination of passive and active intrinsic membrane properties of neurons (Hutcheon and Yarom, 2000).

**Figure 4:**
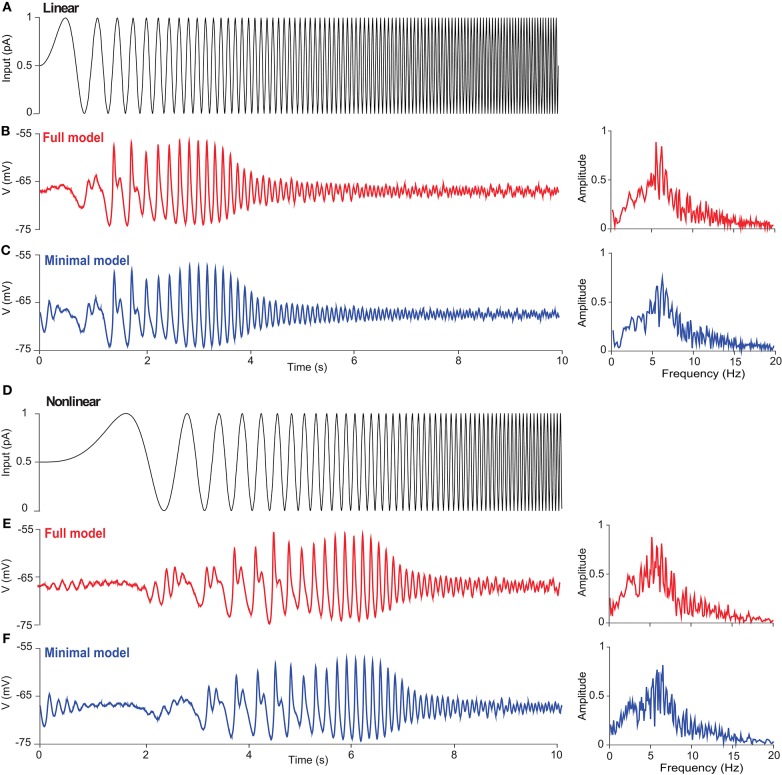
Subthreshold resonance of a UBC in response to sinusoidal inputs with linearly and non-linearly increasing frequency. **(A)** Zap current input from 0 to 10 Hz linearly as *I*_*input*_ = *I*_*amp*_sin(2*f*(*t*)*t*) with *f*(*t*) = *f*_*max*_*t*/*T* at *I*_*amp*_ = 1 pA, *f*_*max*_ = 10 Hz, and *T* = 10 s. **(B)** UBC voltage response triggered by the stimulus given in (A) in the full model. (Right) Fourier transform of UBC response showing the resonance of UBC dynamics with a peak frequency around 6 Hz. **(C)** Similar to **(B)** but simulated with the minimal model. **(D)** Zap current input from 0 to 10 Hz non-linearly as $I_{input}=I_{amp}\sin\left(0.35t^{3}\right)$ at *I*_*amp*_ = 1 pA. **(E,F)** Similar to **(B,C)** showing UBC response and resonance.

Figure 4 shows the subthreshold dynamics of UBC models with linearly (Figures 4A,C) and non-linearly (Figures 4D–F) increasing frequency. There are significant resonance responses for both types of inputs, and the amplitude of membrane potential first increases and then decreases, so that there is a low-frequency (~6 Hz) subthreshold potential resonance in both models, as seen with FFT. Such a subthreshold resonance in UBCs is similar to that in thalamic neurons, which also involves T-type Ca current (Wang et al., 1991). The resonance frequencies of the two UBC models are similar, presumably due to the fact that the T-type Ca current plays an important role in resonance generation. The nature of resonance is intrinsic and independent of whether the input pattern is linear or non-linear. These results reveal that the subthreshold resonance is related to intrinsic properties but independent of the exact types of external inputs.

### 3.4. UBC Response to Current Input

In the classical protocol of *in vivo* recording, when the animal was rotating its head, it was found that the responses of different types of neurons in the vestibular cerebellum presented phase shifts in different degrees (Barmack and Vadim, 2008), where the firing activity of a UBC could be modulated by the input current. When the response is fitted with a sinusoidal function, the phase shifts between UBC response and input current are distributed uniformly, and it has been shown that this uniform distribution contributes to the relaying of input MF information (Zampini et al., 2016).

**Figure 5:**
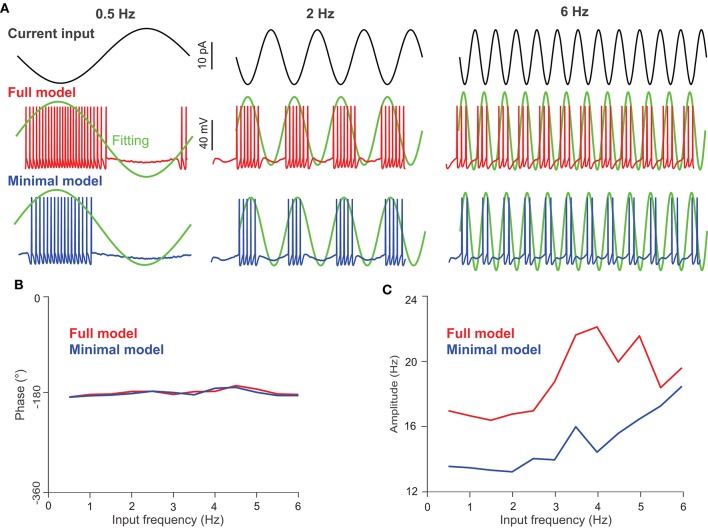
UBC response to oscillating current inputs. **(A)** Response of UBC models to sinusoidal current inputs with 0.5, 2, and 6 Hz frequencies and 10 pA amplitude. (Top) Current inputs of three frequencies. UBC voltage traces obtained from the full (middle) and minimal (bottom) models with fitting curves (green) to compute the phase in degree and amplitude in Hz of UBC responses. The fitting curve was obtained by fitting the instantaneous firing rate of UBC responses. **(B)** The phase shift of UBC response relative to the input phase in the full (red) and minimal (blue) model as a function of modulation frequency. **(C)** Similar to **(B)** but for UBC response amplitude.

We set out to investigate the firing-rate modulation of UBCs using both models with oscillating input currents with a fixed amplitude (10 pA) but at different frequencies; the three typical inputs are shown in Figure 5A (top). Compared to the full model (Figure 5A, middle), the minimal model generates fewer spikes under the same current input (Figure 5A, bottom), presumably due to the absence of L-type and h-type ion channels, which could induce firing via a pacemaking mechanism. After fitting the average UBC firing activity over several input cycles, one can obtain the amplitude and phase shift of modulation with respect to the input. Interestingly, the phase shift of the modulation exhibits a similar behavior for both models (Figure 5B), while the amplitude of the modulation in the full model is larger than that of the minimal model (Figure 5C). This difference is a direct consequence of the higher firing activity in the full model induced by L-type and h-type channels. These results suggest that the phase shifts of the input modulation observed in UBC experiments are not induced by intrinsic ion channel properties, which only contribute to the amplitude of firing rate.

### 3.5. UBC Response to Synaptic Input

So far, we have studied the intrinsic properties of UBCs through different ion channels and the dynamics of UBCs in response to current input. In real scenarios, the firing activity of a single MF encodes the velocity of head rotation in animals (Arenz et al., 2008). Since a single UBC only receives one MF, a mono-connection from MF to UBC makes it easy to study how the firing activity of the UBC is changed by presynaptic inputs.

Recent experiments show that a UBC can embed a large variety of synaptic receptors such that there is a rich diversity of dynamics in its firing activity (Carolina and Trussell, 2015; Zampini et al., 2016). UBC brushes are endowed with different types of synaptic receptors, including the AMPA, NMDA, mGluR1, and mGluR2 receptors (Billups et al., 2002; Zampini et al., 2016). The presynaptic element of these synapses shows the typical mossy fiber terminal but forms an exceptionally extended synaptic contact that can entrap glutamate for several hundred milliseconds, thus enabling unusually long excitatory responses (Ann et al., 2014; Stijn and Zeeuw, 2014; Zampini et al., 2016).

To understand how a UBC may transcode its synaptic inputs, we examined the synaptic response of a UBC by using different types of receptors. Based on the experimental data of UBCs recorded (Zampini et al., 2016), five types of synapses can be modeled: fast AMPA (AMPAf), slow spillover AMPA (AMPAsl), excitatory NMDA and mGluR1, and inhibitory mGluR2 (see section Methods).

**Figure 6:**
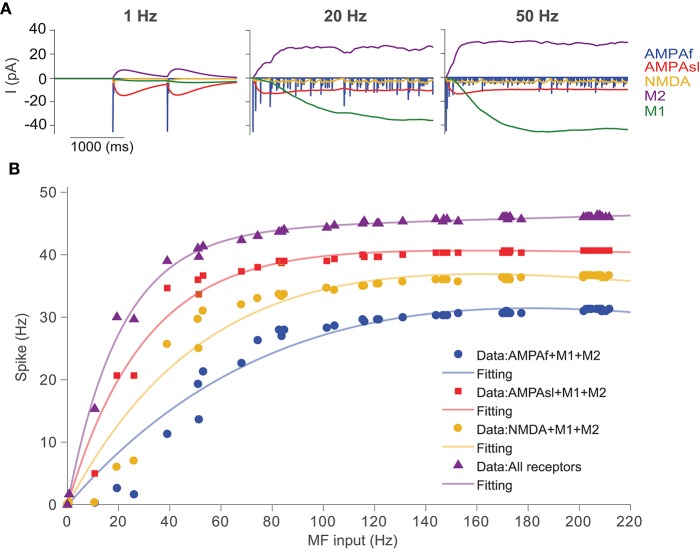
UBC response to synaptic inputs with different types of receptors. **(A)** Receptor dynamics of fast AMPA (AMPAf), slow AMPA (AMPAsl), NMDA, mGluR1 (M1), and mGluR2 (M2) triggered by mossy fiber Poisson spike trains at frequencies of 1, 20, and 50 Hz. **(B)** Spiking output of UBCs with different combinations of synaptic receptors in response to Poisson stimulation of MF inputs.

To simulate the UBC response to the synaptic current of each type of receptor, Poisson stimulations of a single mossy fiber at different frequencies were used, and the UBC voltage was fixed at -57 mV under simulated voltage clamp. We used the minimal UBC model, which has a lower firing activity compared to that of the full model. In this way, one can clearly examine the difference between different receptors to avoid saturation at high firing rate under strong stimulation. Figure 6A shows the simulated excitatory/inhibitory postsynaptic currents of the five individual receptors in the UBC over 4 s using Possion stimuli with the frequency of 1, 20, and 50 Hz, respectively. It can be observed that only the mGluR2 current is positive (inhibitory), while the currents of the other four receptors are negative (excitatory). The output current of the fast AMPA receptor shows a typical behavior of rapid increase and decay. The responses of slow AMPA and mGluR2 are relatively larger and longer than those of the other receptors, so these two play a major role in shaping the response of a UBC to low-frequency stimulation. When Poisson stimulation is changed to higher frequencies, the response currents of these receptors vary significantly. If the stimulation frequency is greater than 20 Hz, the response currents of mGluR1 and mGluR2 increase and saturate over time, and the current after stabilization is much larger than that in the other receptors. Taken together, these synaptic dynamics can individually or together express on different individual UBCs to generate a large variety of temporal dynamics for information relay (Carolina and Trussell, 2015; Zampini et al., 2016).

We then study the input-output relationship of UBC response, which is determined predominantly by the properties of the synapses and their short-term plasticities. The cerebellum can control high-precise motor patterns with millisecond resolution by using a wide range of action potential firing rates. Figure 6B shows the results for the UBC firing rate response with different combinations of synaptic receptors. We measured the spiking response obtained from simulated membrane voltage traces of the UBC model during a single mossy fiber Poisson stimulus with a wide range of frequencies (1-220 Hz) and calculated the UBC output firing frequency, which can be fitted with a double exponential function.

In order to investigate the effects of different receptors on UBC response output, we changed the receptor type of the MF-UBC synapse in the model by combining mGluR1 and mGluR2 with the other three receptors respectively. In this way, the slow dynamics of mGluR1 and mGluR2 can be balanced to amplify the other three receptors, AMPAf, AMPAsl, and NMDA. The results show that the output firing rate is changed when the types of synaptic receptors are altered. A non-linear relationship between output firing frequency and input MF stimulation frequency can be observed. The output discharge frequency increases at the low-frequency stimuli. For higher frequencies, the output discharge frequency increases gradually and then saturates. These results suggest that, for the low frequencies, the slow spillover mechanism of AMPA receptors predominates in coding the frequency of synaptic firing rate. However, all receptors can cooperate to regulate synaptic firing at all frequencies of inputs.

**Figure 7:**
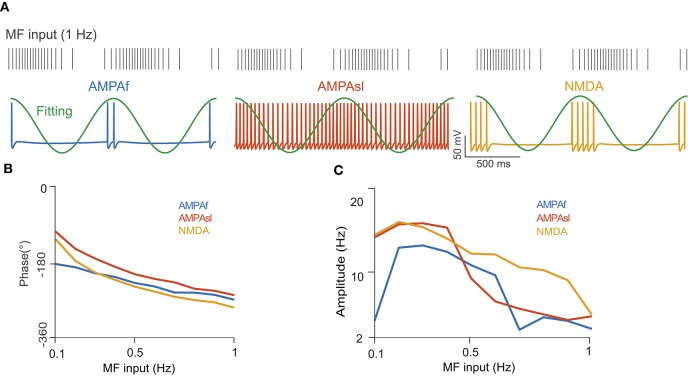
UBC response to MF input with a modulated firing rate. **(A)** (Top) MF spike train input sampled from a modulated sinusoidal firing rate *A* sin(2π*ft*) + *A* with 1 Hz frequency and amplitude *A* = 20 Hz. Vertical ticks indicate spike times. (Bottom) UBC voltage traces simulated with different receptors of AMPAf, AMPAsl, and NMDA, respectively. Fitting curves (green) are sinusoidal functions to compute UBC response modulation in terms of phase shift and amplitude. **(B)** The phase shift of UBC response relative to MF sinusoidal input with three types of receptors as a function of MF input frequencies. **(C)** Similar to **(B)** but for UBC response amplitude.

Next, we examined the firing rate modulation of continuous MF input by different receptors using the full model. For simplicity, we only consider the AMPAf, AMPAsl, and NMDA receptors, as they are shown to generate enough phase shifts for UBCs (Zampini et al., 2016). The UBC voltage traces for different receptors to the same sinusoidal MF input with a modulated firing rate at the frequency of 1 Hz are shown in Figure 7A, where firing rates are significantly different between receptors. Again, the average firing rate of a UBC over several stimulation cycles was fitted by a sinusoidal function to compute the modulation phase (Figure 7B) and amplitude (Figure 7C) in UBC responses. These three types of receptors, AMPAf, AMPAsl, and NMDA, can induce different phase shifts relative to the MF input due to their dynamics occurring at different time scales. These results show that the phase shifts of UBC response relative to the MF input are induced by different types of receptors. Together with the variation of synaptic connection weights, UBCs can relay the MF input over a full spectrum of any direction in the phase space (Zampini et al., 2016).

## 4. Discussion

To understand how information is processed in a brain region, it is crucial to study the intrinsic properties of the neurons within the circuitry. Here, we developed two models for a UBC in the granular layer of the cerebellum based on the experimental data obtained in the literature. We clarified the functional role of the T-type Ca and K channels in shaping the dynamics of UBCs by stability analysis of the resting membrane potential, rebound firing after inhibitory current injection, and subthreshold response resonance. Furthermore, we demonstrated that the rich types of synaptic receptors expressed on UBCs can modulate their strength of firing activity, as well as the amplitude and phase shift of the firing rate relative to presynaptic MF input.

The only known source of excitation for UBCs is their single giant dendritic brush, which can receive the external MFs. The outside input passes into MFs, which can be spontaneously active at high frequency *in vivo* (Arenz et al., 2008) and are thus likely to drive the complex pattern of activity of UBCs observed in experiments. However, UBCs do not discharge spontaneously at rest. We showed, by using mathematical analysis of a reduced minimal model, that the T-type Ca and mGluR2-dependent rectifier K channels play a particularly important role in maintaining the stable state of the resting membrane potential. By comparing simulations with both full and minimal models with theoretical results, we found that the resting potential of a UBC at *V*_*rest*_ = −67 mV yields reasonable values for the conductance parameters *g*_*T*_ and *g*_*K*_, which are consistent with experimental measurements (Diana et al., 2007). These types of analysis and mechanisms may be useful for studying other types of neurons that have no spontaneous activity, for instance, the retinal ganglion cells in the retina of the salamander (Liu and Gollisch, 2015). However, the existence of spontaneous activity, even in the retina, depends on a number of biophysical properties of neurons (Sernagor and Grzywacz, 1999; Margolis and Detwiler, 2007).

UBCs can display rebound burst-firing upon hyperpolarization from their resting membrane potential. Rebound firing has been suggested to play an important role in cerebellar function (Aizenman et al., 1998; Aizenman and Linden, 1999; Kistler and Zeeuw, 2003; Pugh and Raman, 2006) under different *in vitro* and *in vivo* experimental conditions (Karina et al., 2008). In the current study, the experimental and modeling results indicate that UBCs can generate rebound firing with inhibitory current injection, similar to what has been documented in experimental studies (Zampini et al., 2016). In addition, the parameter values of the conductance of the mGluR2-dependent K channel as 0.3 nS and the T-type Ca channel as 3.5 nS are within a reasonable range of experimental conditions. Therefore, the rebound firing of UBCs is most likely mediated by T-type calcium channels, which is consistent with previous findings (Aizenman and Linden, 1999; Molineux et al., 2006).

One of the non-linear features in neuronal computation is that neurons can generate resonance, i.e., enhanced response at a particular preferential frequency (Hutcheon and Yarom, 2000). In the cerebellum, GCs show theta-frequency resonance around 6-8 Hz, which was suggested to be due to the existence of a slow repolarizing K current (D'Angelo et al., 2001). Here, we found that injecting the UBC model with sinusoidal currents of different frequencies can generate resonant responses as well and that subthreshold resonance occurs at a low frequency (~6 Hz). Considering that GCs typically fire at a low frequency (~5–6 Hz), UBCs, as a relay between MFs and GCs, may utilize their subthreshold resonance to maximize the information carried by subthreshold membrane potentials for downstream granule cells (Ann et al., 2014; Zampini et al., 2016).

As well as its intrinsic properties, the UBC has a short dendrite as a presynaptic terminal receiving afferent synaptic input from external MFs. The unique feature of the MF-UBC synapse is that there exists a very extensive synaptic apposition with multiple release sites (Billups et al., 2002) as well as a large variety of synapse receptors, as modeled in the current study. Specifically, UBCs can be classified as ON and OFF types according to their expression of synaptic receptors (Carolina and Trussell, 2015; Zampini et al., 2016). The ON type of UBC shows excitatory responses to glutamate inputs resulting from high expression of AMPARs and mGluR1, whereas the OFF type of UBC shows inhibitory responses to glutamate inputs due to mGluR2 activation. Therefore, receptor heterogeneity can induce a large diversity of dynamics in ring activity, as shown in this work. We found that the slow spillover AMPA receptors, besides typical AMPA and NMDA, play an important role in shaping dynamics, similar to experimental observations (Zampini et al., 2016). Altogether, different synaptic receptors in UBCs seems to play different roles in neuronal computation (Ann et al., 2014; Stijn and Zeeuw, 2014; Carolina and Trussell, 2015; Zampini et al., 2016). The most important part of these computations is to generate different relays, or shifts in terms of phase of MF input, which provide a temporal basis for GCs and Purkinje cells (Ann et al., 2014; Zampini et al., 2016), eventually enabling the fulfillment of the complex computation done by the cerebellum (Raymond and Medina, 2018).

In the current work, due to its simple dendritic structure, we employed a specific type of neuron in the cerebellum, the UBC, to explain how the intrinsic and synaptic properties of a neuron shape diverse behaviors of single neuronal dynamics. The important computational properties for single neurons consist of both intrinsic and synaptic dynamics (Abbott and Regehr, 2004; Harvey et al., 2009). Intrinsic properties play a dominant role in subthreshold response and spiking activity, whereas the synaptic dynamics rely on spikes passing between neurons. Therefore, intrinsic properties can be combined with synaptic dynamics at different time scales to shape diverse neural dynamics (Latorre et al., 2016; Torres et al., 2019). Such interactions become more prominent at the network level (Buonomano and Maass, 2009; Liu and Buonomano, 2009; Liu, 2011). A detailed model with multiple compartments and morphological structure is particularly useful for validating experiments (Subramaniyam et al., 2014). However, to study the network dynamics, modeling of single neurons mostly relies on computationally efficient models similar to the ones used in this work to strike a balance between detail and abstraction (Herz et al., 2006). Eventually, the dynamics of single neurons together with a population of neurons within the same neuronal circuit and across different brain areas form rich dynamical behaviors and computations (Buonomano and Maass, 2009; Yang et al., 2019).

## Data Availability Statement

All datasets generated for this study are included in the article/supplementary material.

## Author Contributions

LA, ZY, and JL: conceptualization and project administration. LA, YT, DW, and SJ: formal analysis. LA, QP, QW, and JL: funding acquisition. LA, YT, and DW: investigation. LA, YT, ZY, and JL: methodology and writing—original draft. YT, DW, and JL: software. LA, QW, and JL: supervision. YT and ZY: validation. YT, ZY, SJ, and JL: visualization.

## Conflict of Interest

The authors declare that the research was conducted in the absence of any commercial or financial relationships that could be construed as a potential conflict of interest.
